# The Influence of Metals in the Development of Microorganisms in Gelatin

**Published:** 1901-11

**Authors:** William H. Potter


### Reviews of Dental Literature.

The Influence of Metals in the Development of Micro-organisms in Gelatin.[1]—The author gives Miller the credit for making the first experiments along this line, and for showing that certain metals had the power of hindering the development of bacteria. His experiments were especially concerned with those metals commonly used as filling-materials.

Behring later confirmed Miller's experiments and extended the knowledge as to the controlling action of gold and other metals upon the growth of various bacteria. Behring held that there was a solution of the metal in the gelatin brought about by the by-products of the growing bacteria, and that only on this supposition could the controlling action of the metals upon the growth of bacteria be explained.

The author starts with the results obtained by Miller and Behring and undertakes a further development of the subject. His method was to place a piece of metal in a culture medium which had been infected with bacteria, and then to note whether the metal hindered or not the growth of bacteria. In this way

---

[1] Ueber Einfluss von Metallen auf die Vermehrung von Mikro-organismen in der Gelatine. Von Dr. Fritz Schenk, Wien. Oesterreichisch-ungarische Vierteljahrsschrift für Zahnheilkunde, Jänner, 1901.

were tested gold, silver, zinc, tin, lead, and iron. Of these metals, lead and tin showed no restraining influence over the growth of bacteria. With iron the effect was very slight. Gold, silver, and zinc, on the other hand, showed a marked influence. The influence of zinc was most marked. Further investigations were made to establish the duration of this action. It was found that at the expiration of twenty-four hours gold still exercised a controlling influence upon an infected gelatin, and that silver held a still greater influence than gold. Lead and tin exercised no influence at all. Iron showed only a very slight influence, while zinc showed the most lasting influence of any of the metals used.

The conclusion which the author draws from the last experiments is that none of the metals exercise a lasting effect upon the growth of bacteria.

After establishing the above results by repeated experiments, a further series was undertaken to determine whether the liquefaction of gelatin by heat was at all influenced by the presence of metals. For this purpose culture gelatin was heated in a water-bath and found to become fluid at 24° to 25° Celsius. But where the metal zinc was present under similar conditions the gelatin did not become fluid until 32° Celsius had been reached. The author feels that he has established the fact that such metals as hinder the growth of bacteria in gelatin also compel a higher temperature for its liquefaction.

In reaching this conclusion experiments were made with zinc, silver, gold, platinum, iron, lead.

The theory that a solution of a part of the metal occurs in the culture gelatin is favored by the author. And in this way a reason is given for the controlling influence which various metals have upon the growth of bacteria, and also for the ability which many possess of requiring a higher temperature for the liquefaction of culture gelatin.

If one seeks to gain some practical points from these very interesting observations upon the influence of metals upon the growth of bacteria in culture gelatin, he will be somewhat disappointed. For while several of the metals—notably zinc, silver, and gold—exercise a decided influence upon bacteria, this influence is only of short duration, and could hardly be considered of much value if relied upon in the case of a filling-material.

William H. Potter.

